# Sulfonic Group Modified Binder Endows Rapid Lithium‐Ion Diffusion for SiO_*x*_ Microparticle Anode

**DOI:** 10.1002/smsc.202300133

**Published:** 2023-11-27

**Authors:** Zheng Weng, Gang Wu, Jiaqi Li, Ying Zhang, Ruifeng Zhang, Ning Zhang, Xiaohe Liu, Chuankun Jia, Gen Chen

**Affiliations:** ^1^ School of Materials Science and Engineering Hunan Provincial Key Laboratory of Electronic Packaging and Advanced Functional Materials Central South University Changsha Hunan 410083 P. R. China; ^2^ Zhongyuan Critical Metals Laboratory and School of Chemical Engineering Zhengzhou University Zhengzhou Henan 450001 P. R. China; ^3^ Institute of Energy Storage Technology Changsha University of Science & Technology Changsha Hunan 410114 P. R. China

**Keywords:** lithium-ion diffusion, SiO_*x*_ microparticle, stress dissipation, sulfonic acid anionic group

## Abstract

Silicon‐based materials have been regarded as the most flourishing anode materials owing to the incomparable specific capacity. However, their commercial application is obstructed by huge volume expansion and particle pulverization, which subsequently lead to the stress concentration and the loss of electrical contact, eventually resulting in poor cycling stability and lousy rate performance. Herein, an ion‐conductive binder with boosted ion‐conductivity is proposed by free radical polymerization between acrylic acid and lithiated 2‐acrylamido‐2‐methyl‐1‐propanesulfonic acid (LiAMPS). With the aid of ample sulfonic acid anionic groups, rapid lithium‐ion diffusion can be achieved to improve the transport kinetics and rate performance. Meanwhile, superior mechanical properties of binder can alleviate the stress concentration to avoid particle pulverization by noncovalent hydrogen bond. The synergistic strategy of constructing lithium‐ion diffusion pathway and alleviating the stress concentration can make a preeminent improvement on Li^+^ diffusion coefficient and maintain a high structural integrity of electrode. Benefiting from the synergistic effect, the SiO_*x*_ microparticle anode delivers a high capacity of 587.8 mAh g^−1^ after 400 cycles at 1C and preeminent rate performance of 648.6 mAh g^−1^ at 5C. Such a synergistic design strategy endows P(AA*‐*co‐LiAMPS) binder with a promising potential for high energy density silicon‐based anodes.

## Graphical abstract

A sulfonic group modified binder with boosted ion conductivity is prepared by free radical polymerization between acrylic acid and lithiated 2‐acrylamido‐2‐methyl‐1‐propanesulfonic acid. With the aid of ample sulfonic acid anionic groups, a rapid lithium‐ion diffusion pathway can be constructed to improve the transport kinetics and rate performance of SiO_*x*_ anode.

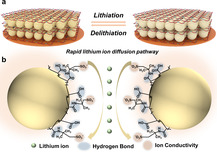

## 1 Introduction

Recently, ultrahigh energy density batteries have a blazing appeal for the emergence of electronic devices without “low battery anxiety”, electric vehicles without “range anxiety”, and large‐scale energy storage systems without “security anxiety”.^[^ ^1^ ^]^ Much efforts have been devoted to the grid‐scale commercialization of lithium‐ion battery (LIB) with rivalrous energy density, superior cycling sustainability, and reliable safety. Silicon has long been highlighted as the most flourishing anode material owing to its incomparable theoretical specific capacity (4200 mAh g^−1^ for Li_4.4_Si), ample crustal abundance, and low lithiation potential (≈0.3 V vs Li^+^/Li).^[^ ^2^ ^]^ Nevertheless, rapid capacity decay and poor cycling stability caused by the infamous volume expansion/contraction (≈380%) during lithiation/delithiation obstruct the next‐generation commercialization of silicon.^[^ ^3^ ^]^ To push forward the large‐scale commercial application of silicon, numerous efforts have been poured into designing silicon composite (Si/C and SiO_*x*_, etc.),^[^ ^4^ ^]^ which is aimed to elevate the electronic conductivity and confine the volume expansion of silicon. However, the detrimental particle pulverization and structural collapse of electrode resulted from huge volume expansion (≈200%) and contraction of SiO_*x*_ are still inevitable.^[^ ^5^ ^]^ Besides, lithium oxide (Li_2_O) and lithium silicate (Li_*x*_Si_*y*_O_*z*_) also generate irreversibly during the first lithium insertion process, which leads to a low initial Coulombic efficiency (ICE).^[^ ^6^ ^]^

To overcome the aforementioned barriers, countless researchers have devoted themselves to designing delicate nanostructure,^[^ ^7^ ^]^ constructing artificial solid electrolyte interface (SEI)^[^ ^8^ ^]^ and exploring advanced binders.[^6^, ^9^] Among these solutions, exploring advanced binders has attracted increasing attention due to its convenience and cost‐effectiveness. Recent auspicious research outcomes have proposed an enormous amount of multifunctional binders for Si‐based anodes, including constructing inorganic‐rich SEI,^[^ ^10^ ^]^ self‐healing,^[^ ^11^ ^]^ energy‐dispersion,^[^ ^12^ ^]^ and interface‐adaptive binders.^[^ ^13^ ^]^ The ionic conductivity of binders also plays a vital role in boosting the transportation of lithium ion.[^11^, ^14^] Therefore, it is extremely urgent to design novel binders with fast lithium‐ion transportation.

Sulfonic group is known as one of the crucial parts in single‐ion conducting polymer electrolyte, which can also be used to endow binders with a strong lithium‐ion conductivity for silicon anode.^[^ ^15^ ^]^ However, few researchers have utilized sulfonic group on promoting the ionic conductivity of binders so far. Herein, we designed a novel ion‐conductive binder by the introduction of negatively charged sulfonic acid anionic group with fast lithium‐ion transportation, thereby decreasing the diffusion impedance of Li^+^ between SiO_*x*_ microparticles. The ion‐conductive binder with abundant sulfonic anionic groups was synthesized for SiO_*x*_ microparticle anode by free radical polymerization between acrylic acid (AA) and lithiated 2‐acrylamido‐2‐methyl‐1‐propanesulfonic acid (LiAMPS). Compared with conventional PAA binders, this ion‐conductive binder with ample sulfonic acid anionic groups can promote the movement of lithium ion along the polymer chains and provide better mechanical properties, consequently improve the overall electrochemical performances of battery.

## 2 Results and Discussions

**Figure 1:**
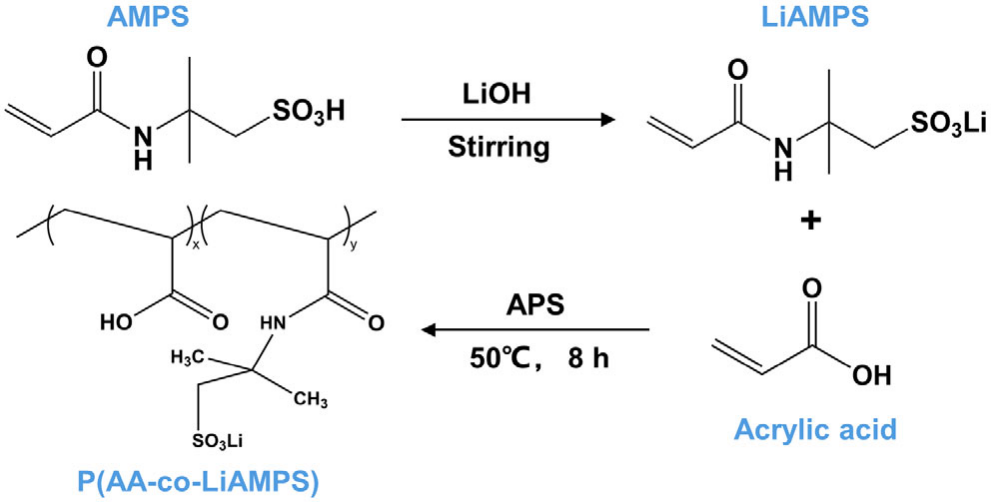
Synthesis of P(AA*‐*co‐LiAMPS) binder by free radical polymerization between AA and LiAMPS monomers.

The P(AA‐co‐LiAMPS) copolymer was prepared by free radical polymerization of AA and LiAMPS, as illustrated in **Figure** **1**. First, AMPS was dissolved in deionized water. Then LiAMPS solution was obtained by adding LiOH into AMPS solution to neutralize the sulfonic acid group of AMPS at the mole ratio of 1:1. AA was dissolved in the prepared LiAMPS solution at the molar ratio of 1:1 (denoted as P11), 2:1 (denoted as P21), 3:1 (denoted as P31 or P(AA‐co‐LiAMPS)), and 4:1 (denoted as P41). Using ammonium persulfate (APS) as an initiator, the free radical polymerization of AA and LiAMPS was triggered under the protection of N_2_.

**Figure 2:**
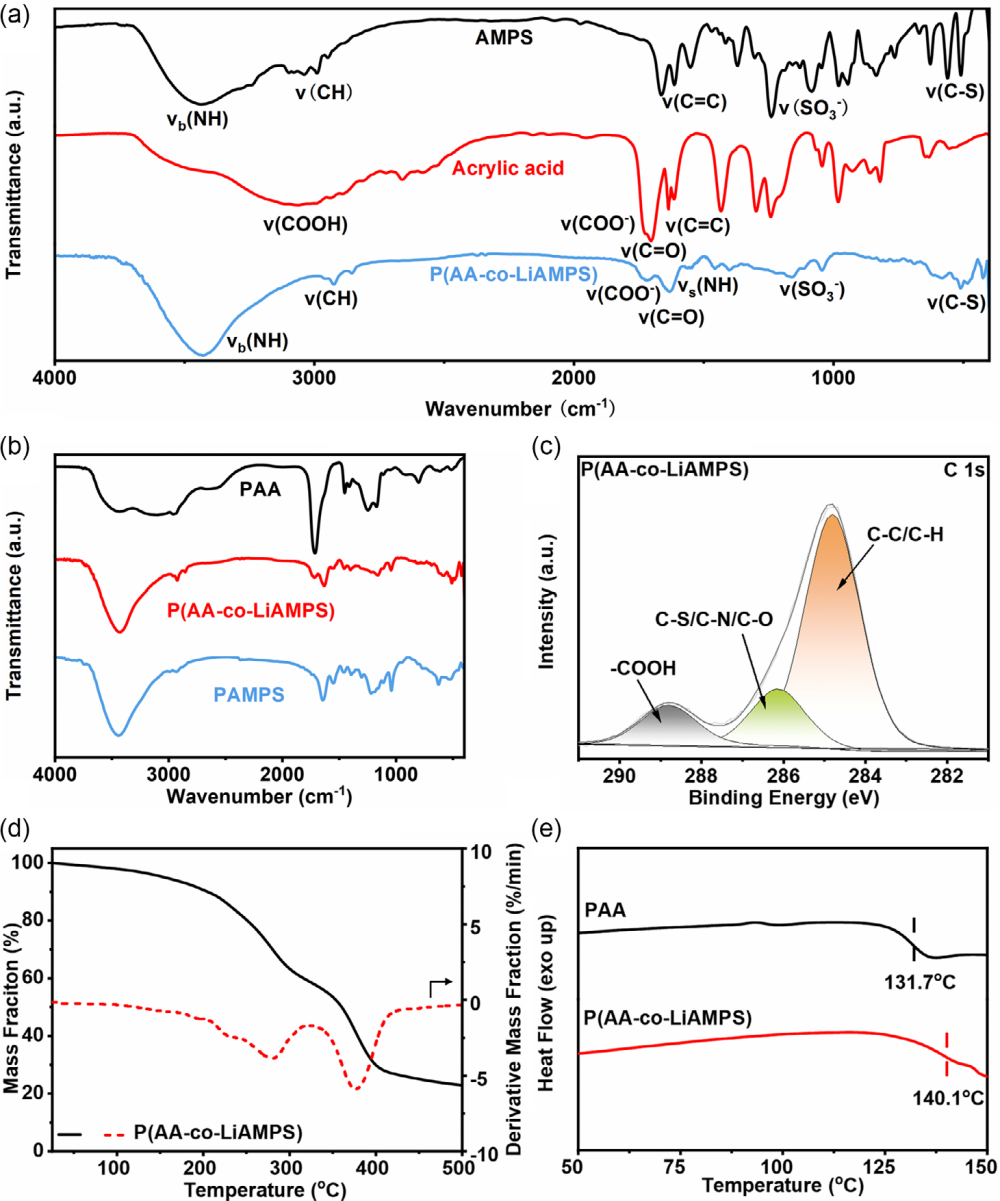
a) FTIR spectra of AA, AMPS, and P(AA*‐*co‐LiAMPS). b) FTIR spectra of PAA, PAMPS, and P(AA*‐*co‐LiAMPS). c) The C 1*s* high‐resolution XPS spectrum of P(AA*‐*co‐LiAMPS). d) The TGA and corresponding DTG curves of P(AA*‐*co‐LiAMPS). e) The DSC curve of PAA and P(AA*‐*co‐LiAMPS).

Inductively coupled plasma optical emission spectrometer was conducted to verify the successful lithiation of LiAMPS. As shown in Table S1, Supporting Information, the content of Li element in P(AA‐co‐LiAMPS) was calculated to be 0.7773 wt%, which is basically consistent with the theoretical content of 0.8140 wt%. Fourier transform infrared spectra (FTIR) is performed to expound the chemical structure of AMPS monomer, AA monomer, and P(AA‐LiAMPS) copolymer. As shown in **Figure** **2**a, the strong absorption peak at 3400 cm^−1^ in the spectrum of AMPS is associated with the bending vibration of imino group (‐NH‐).^[^ ^16^ ^]^ There are several small peaks at about 2900 cm^−1^ ascribed to the stretching vibration of methyl and methylene groups (‐CH_3_ and ‐CH_2_‐).[^16^, ^17^] Besides, two sharp peaks can be observed at 1662 and 1550 cm^−1^ due to the stretching vibration of amide I band (C = O) and amide II band (N‐H), respectively.^[^ ^18^ ^]^ Two characteristic peaks of AMPS appear at around 1240 and 1080 cm^−1^, which correspond to the symmetric and asymmetric stretching vibration of S = O in sulfonic acid group, respectively.^[^ ^16^, ^17^, ^18^ ^-^ ^19^ ^]^ Another characteristic peak located at 500 cm^−1^ represents the existence of C—S bond.[^16^, ^19^] In the spectrum of AA, the broad peak between 2750–3330 cm^−1^ corresponds to the stretching vibration of hydroxyl group in carboxyl group (‐OH in ‐COOH).^[^ ^20^ ^]^ There are two characteristic absorption peaks observed at 1699 and 1612 cm^−1^ which stems from the stretching vibration of C = O and C = C, respectively.[^16^, ^19^] For the P(AA‐co‐LiAMPS) copolymer, the characteristic peak of C = C bond in both monomers at around 1612 cm^−1^ disappears, while the amide I band (C = O), amide II band (N‐H), and sulfonic acid group of LiAMPS, the carboxyl group (‐COOH) of AA can all be observed.

Considering the existence of amide group (‐CONH‐) and sulfonic acid anionic group (‐SO_3_^−^) in LiAMPS, as well as the existence of carboxyl group (COOH) in AA, it is assumed that ample hydrogen bonds will exist between LiAMPS and AA in P(AA‐co‐LiAMPS). Therefore, the homopolymers (PAA and PAMPS) and copolymer (P(AA‐co‐LiAMPS)) of two monomers were characterized by FTIR. As depicted in Figure 2b, the characteristic peak at around 3444 cm^−1^ belongs to the bending vibration of imino group (‐NH‐) in the spectrum of PAMPS. By comparison, the bending vibration peak of imino group (‐NH‐) in the spectrum of P(AA‐co‐LiAMPS) appears at around 3428 cm^−1^, which red‐shifts by 16 cm^−1^ compared with PAMPS. Besides, the amide I band (C = O) at about 1643 cm^−1^ and amide II band (N‐H) at about 1552 cm^−1^ in PAMPS slightly red‐shift to 1632 and 1550 cm^−1^ in P(AA‐LiAMPS), respectively. The asymmetric stretching vibration of ‐SO_2_ in sulfonic acid group also red‐shifts from 1042 cm^−1^ in PAMPS to 1038 cm^−1^ in P(AA‐LiAMPS), while a broader characteristic peak appears at around 1240 cm^−1^ belonging to the symmetric stretching vibration of ‐SO_2_ in sulfonic acid group. Compared to homopolymers, the red‐shift effect and broadening effect of corresponding characteristic peaks at copolymer imply the existence of hydrogen bonds among amide group (‐CONH‐) and sulfonic acid anionic group (‐SO_3_^−^) in LiAMPS of AMPS, as well as carboxyl group (COOH) of AA, which may strengthen the interaction among polymer chains and result in a more stable structure of binder. The slight red‐shift effect and broadening effect of characteristic peaks (COOH, C = O, ‐NH‐, ‐SO_3_^−^) can also be observed in the spectra of pure P(AA‐co‐LiAMPS) and P(AA‐co‐LiAMPS) combined with SiO_*x*_ as binder (Figure S3, Supporting Information), which will also intensify the interaction between binder and SiO_*x*_.

As illustrated in Figure 2c, X‐ray photoelectron spectroscopy (XPS) was conducted to explore the functional groups of P(AA‐co‐LiAMPS). The C 1s spectrum clearly shows three peaks at around 284.8, 286.13, 288.8 eV, corresponding to C‐C/C‐H, C‐S/C‐N/C‐O, and ‐COOH, which identifies the existence of AA in the polymer.^[^ ^21^ ^]^ As for the S 2p spectrum (Figure S4, Supporting Information), the signals of S 2*p*1/2 and S 2*p*3/2 are ascribed to sulfonic acid anion (SO_3_^−^), proving the existence of LiAMPS in the polymer.^[^ ^22^ ^]^^1^H NMR spectra was employed to probe the proton resonances. As shown in Figure S5, Supporting Information, the peak located at *δ* = 1.47 ppm is ascribed to the methyl hydrogen (CH_3_, labeled with “c”) of LiAMPS in the spectra of P(AA‐co‐LiAMPS).^[16a]^ The peaks between *δ* = 1.65 ppm and *δ* = 1.77 ppm belong to methylene (CH_2_, labeled with “a”) of PAA and P(AA‐co‐LiAMPS).^[^ ^23^ ^]^ The peaks of methine (CH, labeled with “b”) in PAA and P(AA‐co‐LiAMPS) appear at between *δ* = 1.95 ppm and *δ* = 2.38 ppm.^[^ ^20^ ^]^ Besides, compared to PAA, a new peak located at *δ* = 3.34 ppm (methylene, CH_2_, labeled with “d”) can be observed in the spectra of P(AA‐co‐LiAMPS) due to the strong electron‐withdrawing ability of sulfonic acid anionic group.^[16a]^ As shown in Figure S6, Supporting Information, the prepared P(AA‐co‐LiAMPS) copolymer possesses an accurately controlled molecular weight. The P(AA‐co‐LiAMPS) copolymer has a Mn of 178 249 g mol^−1^ and a Mw of 335 304 g mol^−1^, which corresponds to a relatively low polydispersity index of 1.88. In conclusion, the molecular architecture of polymers is precisely controlled by free radical polymerization reaction.

Thermogravimetric analysis (TGA) was conducted to survey the thermal stability of P(AA‐co‐LiAMPS). As depicted in Figure 2d, P(AA‐co‐LiAMPS) shows classical two‐stage degradation behavior.^[16a]^ With the temperature elevated above 150 °C, the copolymer displays slight weight drop owing to a loss of moisture. When the temperature is elevated above 200 °C, the copolymer experiences the first‐stage degradation. The anhydride reaction between carboxyl groups will not end until the temperature achieves above 300 °C.^[^ ^20^ ^]^ The second‐stage degradation between 300 and 420 °C originates from the decomposition of sulfonic acid group.^[^ ^17^ ^]^ As observed from TGA curve, the copolymer maintains a relatively wide‐temperature thermal stability before 150 °C, which will be beneficial to avoid the security anxiety of LIBs. Differential scanning calorimeter (DSC) was employed to investigate the glass transition temperature (*T*_g_) of polymer. As shown in Figure 2e, PAA exhibits a lower glass transition temperature (*T*_g_) value of 131.7 °C, while the *T*_g_ of P(AA‐co‐LiAMPS) is up to 140.1 °C. The aforementioned result just implies the existence of hydrogen bond between AA and LiAMPS in the copolymer molecular chains, which impedes the internal rotation of chemical bonds and ultimately brings about a higher glass transition temperature.^[16a]^

X‐ray diffraction (XRD) was performed to compare the crystallinity of two homopolymers and copolymer. Figure S7, Supporting Information, shows the XRD patterns of PAA, PAMPS, and P(AA‐co‐LiAMPS) from 5 to 80°. PAA displays a strong peak at about 2*θ* = 19.91°, corresponding to a layer space of 0.4454 nm. The XRD patterns of PAMPS show a characteristic peak at around 2*θ* = 20.19°, which is assigned to a layer space of 0.4393 nm. It can be observed that a characteristic peak locates at 2*θ* = 22.53° in the XRD patterns of P(AA‐co‐LiAMPS), representing a layer space of 0.3942 nm. Besides, PAA also exhibits a broad peak at 2*θ* = 37.63°, which almost disappears in the XRD patterns of P(AA‐co‐LiAMPS). The shift of characteristic peaks and decline of crystallinity in P(AA‐co‐LiAMPS) are ascribed to closer molecular packing,^[^ ^24^ ^]^ indicating the presence of strong intermolecular interaction represented by hydrogen bond between functional groups in AA and LiAMPS.

**Figure 3:**
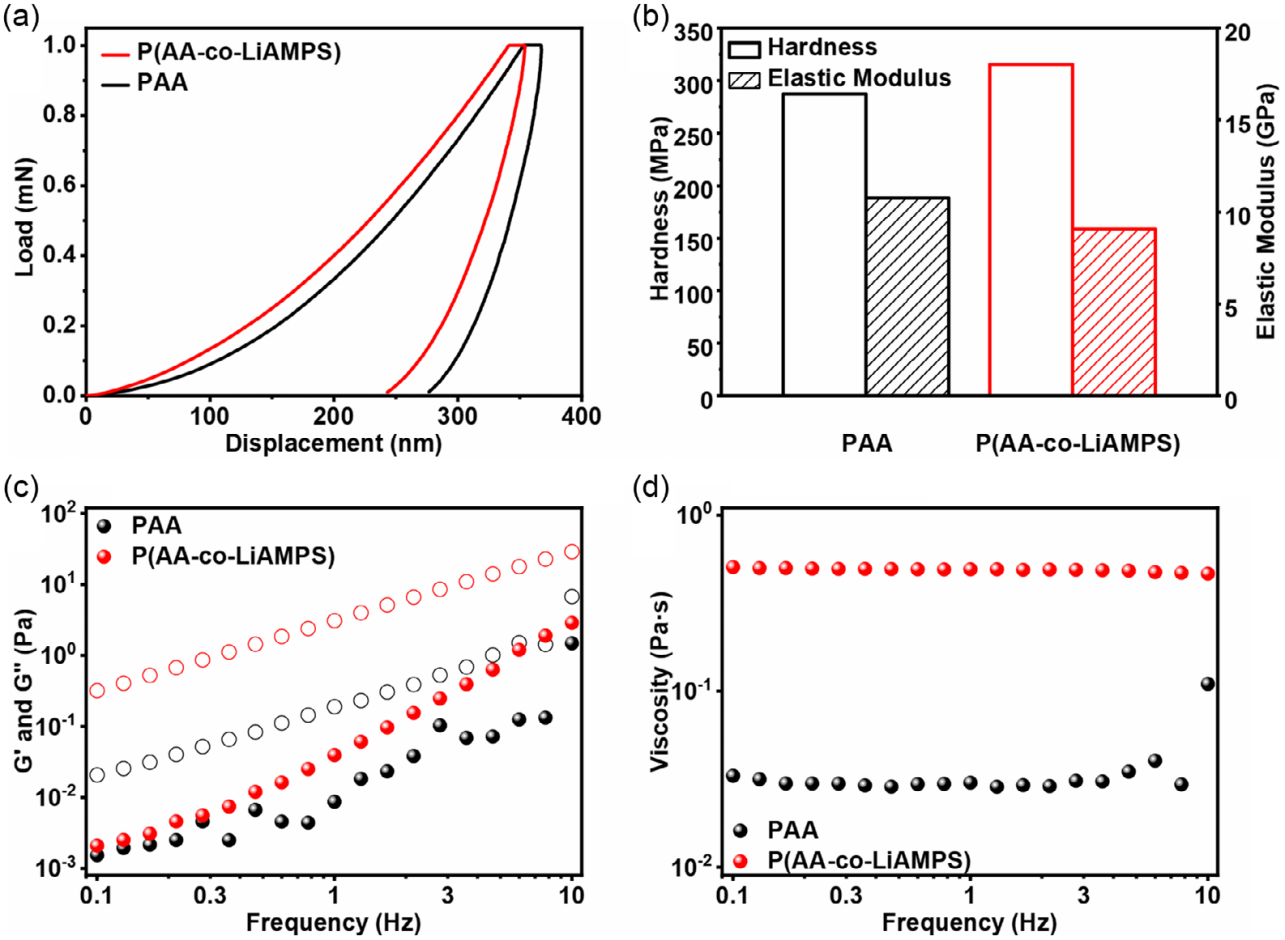
a) The load–displacement curves of PAA and P(AA*‐*co‐LiAMPS) films in nano‐indentation test and b) corresponding hardness and elastic modulus. c) The rheological properties of 5 wt% PAA and P(AA*‐*co‐LiAMPS) aqueous solution. d) Complex viscosities of 5 wt% PAA and P(AA*‐*co‐LiAMPS) aqueous solution.

**Figure** **3**a shows the load–displacement curves of two polymer films in nano‐indentation test and corresponding hardness and elastic modulus. As shown in Figure S8a,b, Supporting Information, the integral area between loading curve and indentation depth represents the total work (*W*_total_) done by the indenter during this loading process, while the integral area between unloading curve and indentation depth stands for the elastic work (*W*_elast_) done by the indenter during this loading process. As shown in Table S2, Supporting Information, the ratio (labeled as “nit”) of elastic work to total work is usually applied to indicate the elasticity of polymers. The value of nit for PAA is calculated to be 22.43%, while the nit value of P(AA‐co‐LiAMPS) is 27.36%. The higher nit value indicates that P(AA‐co‐LiAMPS) possesses better elasticity and deformation recovery ability upon undertaking external compressive deformation. The hardness and elastic modulus of P(AA‐co‐LiAMPS) film are calculated to be 315.3 MPa and 9.05 GPa, respectively (Figure 3b). Conversely, PAA possesses a lower hardness of 287.04 MPa and a higher elastic modulus of 10.76 GPa. The lower elastic modulus and higher hardness of P(AA‐co‐LiAMPS), owing to the reconstruction of noncovalent hydrogen bond between polymer chains under external compressive strain, suggest that P(AA‐co‐LiAMPS) owns slightly lower tolerance to compressive deformation but more excellent resilience than PAA while facing enormous volume contraction of SiO_*x*_.

(1) $$\text{nit}=\frac{W_{\text{elast}}}{W_{\text{elast}}+W_{\text{plast}}}$$

The introduction of AMPS may have a great influence on rheological properties of P(AA‐co‐LiAMPS) polymer molecules, which can be verified by rheometer based on 5 wt% binder aqueous solution (Figure 3c). The storage modulus (*G*′) and loss modulus (*G*″) of two polymers aqueous solutions are two significant indicators to reflect the capability of recovering invertible elastic deformation and dissipating irreversible viscous deformation, respectively. At the same concentration, P(AA‐co‐LiAMPS) solution exhibits higher *G*′ and *G*″ than PAA solution, implying better elastic deformation recovery and viscous deformation dissipation, which may profit from gradient dissociation reconstruction of strong hydrogen bond between functional groups upon withstanding shearing stress. Figure 3d exhibits the complex viscosity of 5 wt% two polymers aqueous solution. P(AA‐co‐LiAMPS) always displays higher complex viscosity from low frequency (0.5074 Pa·s at 0.1 Hz) to high frequency (0.4652 Pa·s at 10 Hz), while the complex viscosity of PAA was as low as 0.0331 Pa·s at 0.1 Hz and 0.11 Pa·s at 10 Hz. Generally speaking, the viscosity of P(AA‐co‐LiAMPS) is nearly 15 times higher than that of PAA, implying that P(AA‐co‐LiAMPS) endures stronger internal friction force, which proves the remarkable enhancement of interaction between polymer molecules derived from hydrogen bond. The adhesion ability of binders is of vital importance to avoid active substance spalling and maintain the integrity of electrode. Typical 180° peeling tests of SiO_*x*_ electrodes with different binders were carried out to evaluate the adhesion ability of binders. As shown in Figure S9, Supporting Information, the SiO_*x*_@P(AA‐co‐LiAMPS) electrode exhibits higher peeling force during the whole peeling process, which is almost 9 times that of SiO_*x*_@PAA electrode, suggesting superior adhesive ability of P(AA‐co‐LiAMPS) binder than PAA binder.

Prior to investigating the influence of prepared binders on battery cycle performance, the Li||Cu half cells were assembled to evaluate the electrochemical stability of P(AA‐co‐LiAMPS) binder. As depicted in Figure S10, Supporting Information, the cyclic voltammetry (CV) curve of P(AA‐co‐LiAMPS)@Cu shows a shape similar to that of bare Cu without redundant peaks, which verifies that P(AA‐co‐LiAMPS) possesses superior electrochemical stability within the working potential range of SiO_*x*_ anode. Besides, large quantities of stochastic peaks (current spikes) derived from occasional factors can be obviously observed in the CV curve of bare Cu,^[^ ^25^ ^]^ while P(AA‐co‐LiAMPS) immensely inhibits the emergence of current spikes, contributing to a more stable electrochemical environment in battery. To gain the optimal mole ratio of P(AA‐co‐LiAMPS) binders, the cycling performances of SiO_*x*_ anodes within 20 cycles with different binders were investigated. As illustrated in Figure S11, Supporting Information, after 20 cycles at the rate of 1C, P31 anode exhibits highest capacity among all anodes, which verifies the optimal mole ratio of AA monomer and LiAMPS monomer in copolymer binders is 3:1. From the perspective of molecular structure, amide group and sulfonic acid anionic group provide hydrogen bond interaction and ion conductivity for P(AA‐co‐LiAMPS) binders, respectively, which is of assistance in improving battery performance. However, tremendous steric hindrance caused by methyl in LiAMPS immensely weakens the interaction between binders and SiO_*x*_, hindering the improvement of battery performance. Limited by the compromise of hydrogen bond, ion‐conductivity, and steric hindrance, P(AA‐co‐LiAMPS) binders with higher molar ratio (P41) exhibits inferior performance to P31. As a result, all investigations based on experimental group involved in the later part all refer to P31 (denoted as P(AA‐co‐LiAMPS)).

**Figure 4:**
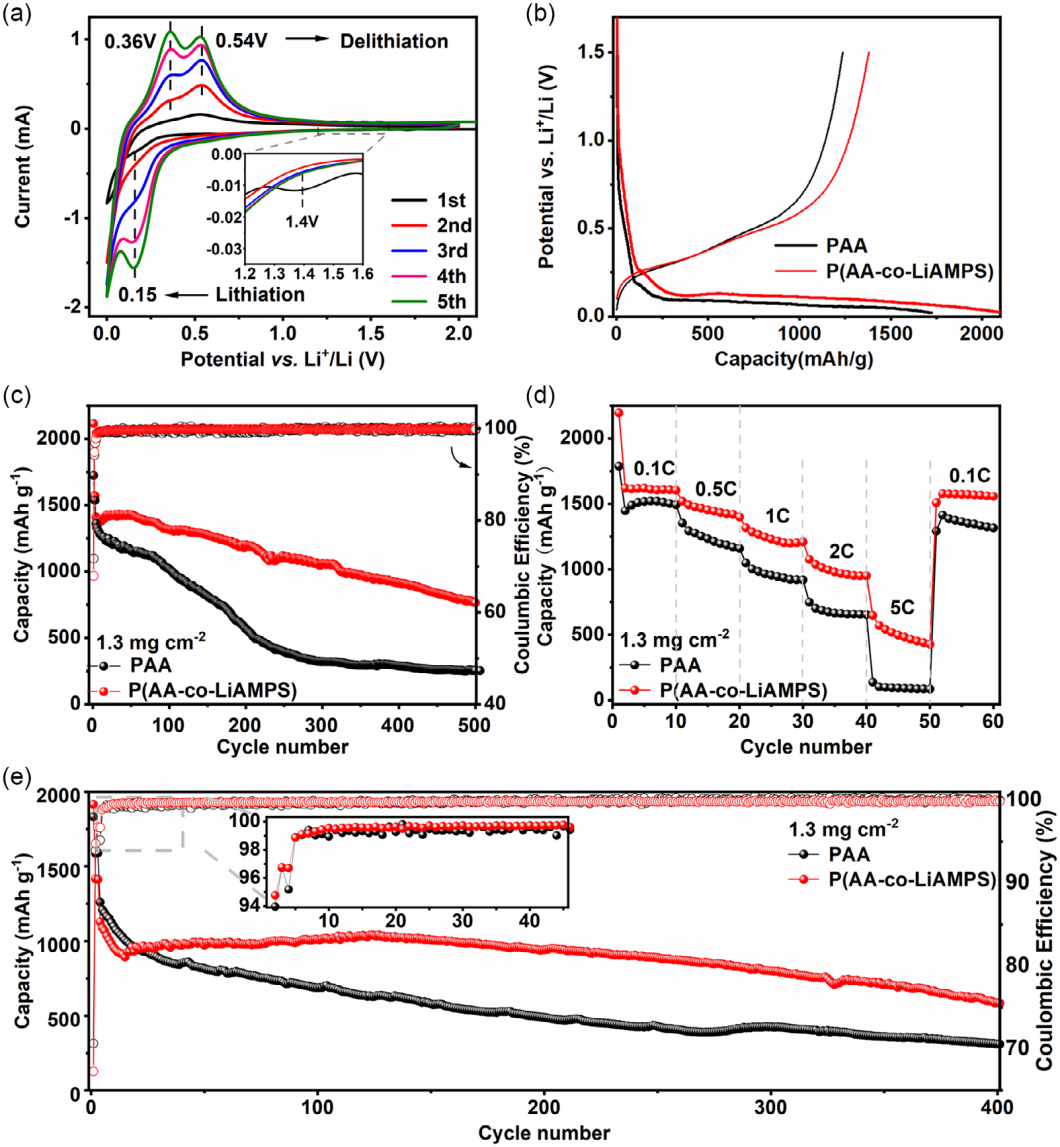
a) CV profiles of SiO_*x*_@P(AA*‐*co‐LiAMPS) anode. b) Galvanostatic charge–discharge profiles of different anodes at the first cycle. c) Cycling performances of different anodes at 0.5C. d) Rate performances of different anodes. e) Cycling performances of different anodes at 1C.

CV was performed to prove the effect of P(AA‐co‐LiAMPS) binder on the electrochemical behavior of SiO_*x*_ microparticles. As shown in **Figure** **4**a, a broad cathodic peak can be visibly observed at approximately 1.4 V due to the formation of SEI derived from electrolyte decomposition during the first lithiation process. The broad peak disappears at the subsequent cycles, which indicates that a stable SEI is established on the surface of SiO_*x*_ microparticles. Another cathodic peak that appears at 0.15 V is associated with the formation of Li–Si alloy. Two broad anodic peaks appear at around 0.36 and 0.54 V, corresponding to the delithiation process of Li–Si alloy. Besides, the increasing peak current verifies the excellent infiltration of electrolyte to electrode and subsequently full activation of SiO_*x*_ microparticles. The superior cycling performance of SiO_*x*_@P(AA‐co‐LiAMPS) anode was evaluated at 0.5C. Figure 4b shows that SiO_*x*_@PAA anode exhibits a lower initial discharge specific capacity of 1724 mAh g^−1^ with an ICE of 71.75%. SiO_*x*_@P(AA‐co‐LiAMPS) anode displays an immensely excellent initial discharge specific capacity of 2114.86 mAh g^−1^ but a slightly lower ICE of 67.97%. The higher ICE of SiO_*x*_@PAA anode may be ascribed to stronger covalent interaction between SiO_*x*_ microparticles and binder derived from more carboxyl groups in PAA, which maintains a more compact electrode structure in the first few cycles. However, single covalent interaction is insufficient to maintain the cycling stability of SiO_*x*_@PAA anode due to the inadequacy of elasticity after only 100 cycles (Figure 4c). Even worse, only an extremely low capacity of 556 mAh g^−1^ was remained for SiO_*x*_@PAA anode after 200 cycles. Particularly, SiO_*x*_@P(AA‐co‐LiAMPS) anode exhibits a comforting capacity of 764.6 mAh g^−1^ with an average CE up to 99.7% after 500 cycles. Regrettably, SiO_*x*_@PAA anode just maintains a capacity of 256.1 mAh g^−1^ after 500 cycles, with a slightly lower average CE of 99.6%.

Apart from superior cycling performance, extraordinary rate performance of SiO_*x*_@P(AA‐co‐LiAMPS) anode was further expounded at different current densities (Figure 4d). SiO_*x*_@P(AA‐co‐LiAMPS) anode exhibits superior capacities of 1621.6 (0.1C), 1519.1 (0.5C), 1317.4 (1C), 1076.1 (2C), and 648.6 mAh g^−1^ (5C). The reversible capacity of SiO_*x*_@P(AA‐co‐LiAMPS) anode rebounds to 1508.8 mAh g^−1^ after the charging/discharging rate returns to 0.1C. The preeminent rate performance of SiO_*x*_@P(AA‐co‐LiAMPS) anode may be ascribed to rapid lithium‐ion diffusion pathway provided by sulfonic acid anionic groups and reversible noncovalent hydrogen bond derived from interaction among amide groups, carboxyl groups, and SiO_*x*_ microparticles. However, SiO_*x*_@PAA anode only delivers poor capacities of 1048.6 (1C), 749.1 (2C), and 139.2 mAh g^−1^ (5C), respectively. Even worse, when the rate returns to 0.1C, SiO_*x*_@PAA anode remains a barely satisfactory capacity of 1292.9 mAh g^−1^. The mediocre rate performance of SiO_*x*_@PAA anode mainly results from particle pulverization and structural collapse of electrode due to the breakage of covalent bond between PAA binder and SiO_*x*_ microparticles after suffering from grievous volume expansion and contraction.

To highlight the long‐term cycling stability of binder, the long cycle test of SiO_*x*_@P(AA‐co‐LiAMPS) anode was conducted at 1C (Figure 4e). After three cycles of activation at 0.1C, SiO_*x*_@PAA anode exhibits a superior capacity compared to SiO_*x*_@P(AA‐co‐LiAMPS) anode during the first 30 cycles. However, SiO_*x*_@PAA anode suffers violent capacity decay during the next 400 cycles, while SiO_*x*_@P(AA‐co‐LiAMPS) anode maintains higher capacity. Besides, the CE of SiO_*x*_@P(AA‐co‐LiAMPS) anode rapidly ascends above 99% only after 10 cycles, while SiO_*x*_@PAA anode delivers an acutely fluctuant CE during the initial 30 cycles. After 400 cycles, SiO_*x*_@P(AA‐co‐LiAMPS) anode exhibits a preeminent capacity of 587.8 mAh g^−1^ with a capacity retention of 52.05% relative to the capacity after activation, while SiO_*x*_@PAA anode only maintains a capacity of 311.5 mAh g^−1^ with an awful capacity retention of 24.72% relative to the capacity after activation. Furthermore, Galvanostatic charge–discharge profiles of SiO_*x*_ anodes with different binders during the first 200 cycles were expounded in Figure S12, Supporting Information. SiO_*x*_@PAA anode exhibits a continuous capacity decay, while SiO_*x*_@P(AA‐co‐LiAMPS) anode maintains an almost constant capacity of 1000 mAh g^−1^ after activation, demonstrating the extraordinary long‐term cycling performance. To highlight the potential of P(AA‐co‐LiAMPS) binder for commercial application, the cycling stability of high‐loading SiO_*x*_ electrodes using P(AA‐co‐LiAMPS) as binder also was investigated at 1C (Figure S13, Supporting Information). After 100 cycles, the SiO_*x*_@P(AA‐co‐LiAMPS) anode with the loadings of 2.28 and 2.86 mg cm^−2^ exhibit relatively high areal capacities of 2.13 and 1.91 mAh cm^−2^, respectively, corresponding to the capacity retentions of 73.91% and 55.22% relative to the capacity after activation. Even at a higher loading of 3.95 mg cm^−2^, the SiO_*x*_@P(AA‐co‐LiAMPS) anode still maintains a high areal capacity of 2.74 mAh cm^−2^ with a capacity retention of 60.15% relative to the capacity after activation. Although the electrode will suffer from greater volume change under high loading, which is more likely to result in extremely serious capacity decay, SiO_*x*_@P(AA‐co‐LiAMPS) electrode also achieves relatively stable operation benefiting from the high ion conductivity and resilience of binder.

**Figure 5:**
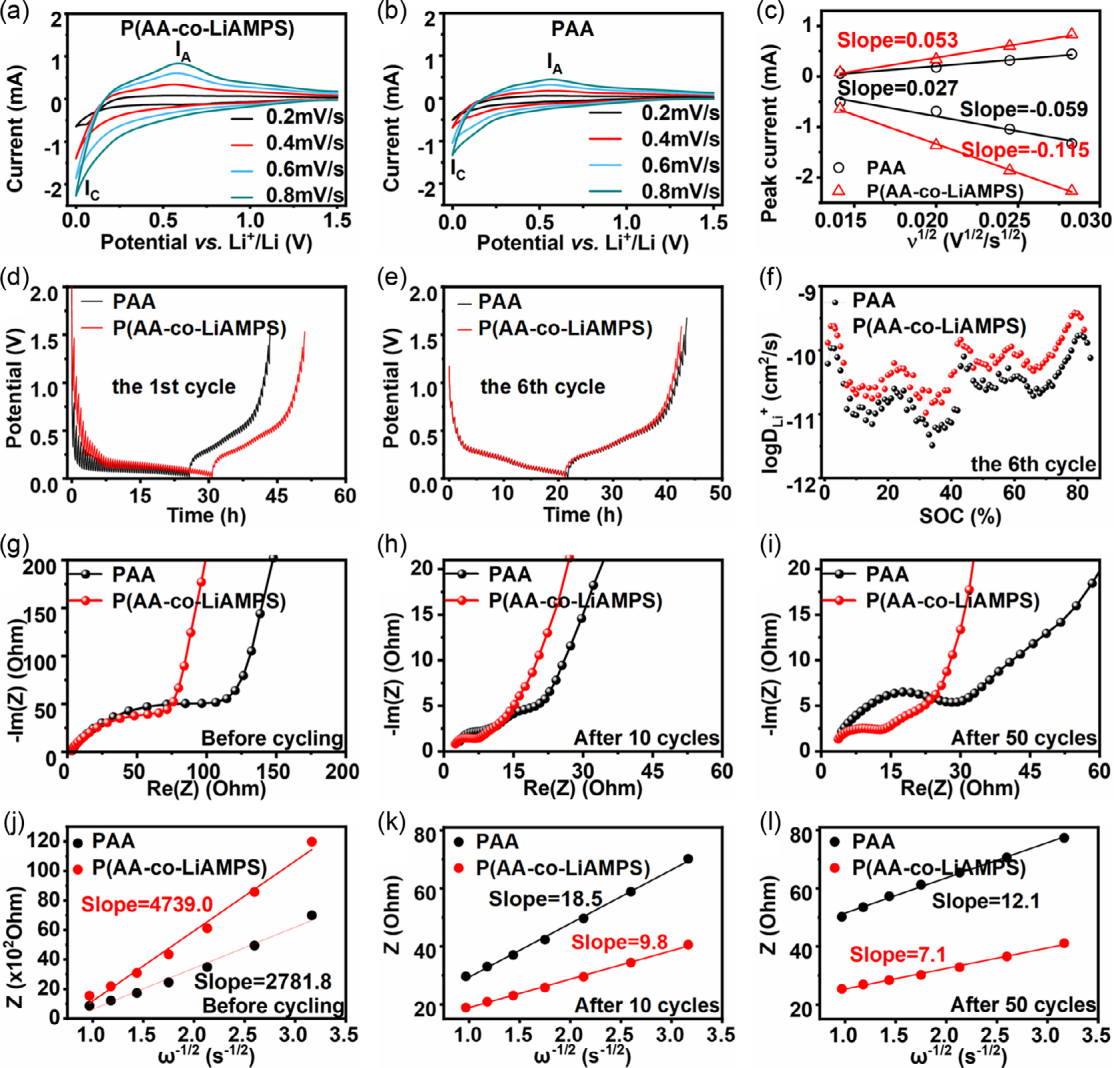
CV profiles of a) SiO_*x*_@PAA anode and b) SiO_*x*_@P(AA*‐*co‐LiAMPS) anode at different scan rates. c) *I*_p_‐*ν* ^1/2^ curves of SiO_*x*_@PAA and SiO_*x*_@P(AA*‐*co‐LiAMPS) anodes. GITT curves of SiO_*x*_@PAA and SiO_*x*_@P(AA*‐*co‐LiAMPS) anodes during d) the first cycle and e) the sixth cycle. f) log*D*_Li_^+^‐SOC curves of SiO_*x*_@PAA and SiO_*x*_@P(AA*‐*co‐LiAMPS) anodes. EIS curves of SiO_*x*_@PAA and SiO_*x*_@P(AA*‐*co‐LiAMPS) anodes g) before cycling, h) after 10 cycles and i) after 50 cycles. *Z*–*ω* ^-1/2^ curves of SiO_*x*_@PAA and SiO_*x*_@P(AA*‐*co‐LiAMPS) anodes j) before cycling, k) after 10 cycles and l) after 50 cycles.

To explore the relationship between superior electrochemical performance of SiO_*x*_@P(AA‐co‐LiAMPS) anode and high ion conductivity improved by LiAMPS monomer, the Li^+^ diffusion coefficients (*D*_Li_^+^) of SiO_*x*_ anode with PAA and P(AA‐co‐LiAMPS) binders were analyzed by CV under different scan rates, Galvanostatic intermittent titration (GITT) test during the first cycle and the sixth cycle and electrochemical impedance spectroscopy (EIS) before cycling, after 10 cycles and after 50 cycles. **Figure** **5**a,b exhibits the CV curves of SiO_*x*_@PAA and SiO_*x*_@P(AA‐co‐LiAMPS) anodes at the scan rates of 0.2, 0.4, 0.6, and 0.8 mV s^−1^. SiO_*x*_@P(AA‐co‐LiAMPS) anode displays a similar CV curve shape with SiO_*x*_@PAA anode, while the former exhibits a peak current nearly twice larger than the latter. As shown in Figure 5c, the anodic peak current (*I*_A_) is positively correlated with the square root of scanning rates (*ν* ^1/2^), while the cathodic peak current (*I*_C_) presents a negative correlation with *ν* ^1/2^. The cracking linear relationship between peak currents and *ν* ^1/2^ can be used to evaluate the *D*_Li_^+^ of SiO_*x*_ anode according to Randles–Sevcik equation.^[^ ^15^ ^]^

(2)

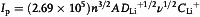




Typically, *I*_p_ represents the anodic or cathodic peak current (A); n refers to the number of electron transfer in lithiation and delithiation process; *A* is obtained by the calculating the electrode area (cm^2^); ν represents the scanning rate (V s^−1^); *C*_Li_^+^ refers to the concentration (mol cm^−3^) of Li^+^ in the electrolyte; and the electron‐transfer number n is calculated based on the element content of SiO_*x*_ microparticles. As shown in Figure S14, Supporting Information, SiO_*x*_@PAA anode exhibits a low *D*_Li_^+^ (0.83 × 10^−9^ and 3.97 × 10^−9^ cm^2^ s^−1^ for delithiation and lithiation process, respectively), which is precisely the reason for its poor rate performance. However, SiO_*x*_@P(AA‐co‐LiAMPS) anode displays a *D*_Li_^+^ (3.20 × 10^−9^ and 15.08 × 10^−9^ cm^2^ s^−1^ for delithiation and lithiation process, respectively) nearly four times larger than SiO_*x*_@PAA anode. Such a result implies that better lithium‐ion diffusion kinetics exists in SiO_*x*_@P(AA‐co‐LiAMPS) anode due to the rapid lithium‐ion diffusion pathway provided by LiAMPS.

Furthermore, considering the variability of lithium‐ion diffusion kinetics at different stages of charging and discharging, GITT was conducted to investigate the diffusion kinetics of lithium ions during the process of charging and discharging in real time according to Fick's second law (Figure 5d,e).^[^ ^26^ ^]^

(3)

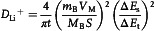




In general, *t* refers to the titration time (s); *m*_B_, *V*_M_, *M*_B_ represent the mass (g), molar volume (cm^3^ mol^−1^), and molar mass (g mol^−1^) of different SiO_*x*_ anodes, respectively; Δ*E*_s_ is the voltage variation induced by titration (V); Δ*E*_t_ represents the voltage variation in single titration period (V); and *S* is the electrode area (cm^2^). As shown in Figure S15, Supporting Information, SiO_*x*_@PAA anode displays a *D*_Li_^+^ roughly the same as SiO_*x*_@P(AA‐co‐LiAMPS) anode at the first cycle, which is mainly due to the inadequate activation of SiO_*x*_ anode. However, after five cycles, SiO_*x*_@PAA anode significantly exhibits a much lower *D*_Li_^+^ than SiO_*x*_@P(AA‐co‐LiAMPS) anode (Figure 5f). Much higher *D*_Li_^+^ of SiO_*x*_@P(AA‐co‐LiAMPS) anode clearly demonstrates its rapid lithium‐ion diffusion kinetics.

The resistance of SiO_*x*_ anodes before and after cycling was conducted by EIS to analyze the transportation kinetics of lithium ion. The equivalent circuit model of SiO_*x*_ anode was illustrated in Figure S16, Supporting Information, and the corresponding calculated resistances were depicted Table S3, Supporting Information. Before cycling, all SiO_*x*_ anodes display one semicircle and one straight line in the fitted EIS curves (Figure 5g), which correspond to the resistances of electrical transfer (*R*_ct_) and ion diffusion (*Z*_w_), respectively. However, SiO_*x*_@PAA anode exhibits a much larger semicircle diameter than SiO_*x*_@P(AA‐co‐LiAMPS) anode, which demonstrates that SiO_*x*_@PAA anode possesses higher *R*_ct_ (121.8 Ω) than that of SiO_*x*_@P(AA‐co‐LiAMPS) anode (78.3 Ω). After 10 cycles, an extra semicircle occurred in both the fitted EIS curves of two SiO_*x*_ anodes (Figure 5h). The former semicircle at high‐frequency region is attributed to the resistances of SEI layer (*R*_SEI_), while the latter semicircle at middle frequency region is related to the electrical transfer (*R*_ct_). Besides, the slope of straight line at low‐frequency region corresponds to the diffusion coefficients of Li^+^. As a result, SiO_*x*_@P(AA‐co‐LiAMPS) anode possesses lower *R*_SEI_ (2.8 Ω) and *R*_ct_ (5.8 Ω), while SiO_*x*_@PAA anode presents relatively high *R*_SEI_ (4.7 Ω) and *R*_ct_ (9.2 Ω) nearly two times larger than the former. After 50 cycles, SiO_*x*_@P(AA‐co‐LiAMPS) anode exhibits lower *R*_SEI_ (3.5 Ω) and *R*_ct_ (13.4 Ω), while SiO_*x*_@PAA anode presents much higher *R*_SEI_ (11.7 Ω) and *R*_ct_ (30.3 Ω) (Figure 5i). In conclusion, SiO_*x*_@P(AA‐co‐LiAMPS) anode shows much lower resistances (*R*_SEI_ and *R*_ct_) and slower increasing tendency than SiO_*x*_@PAA anode whenever before and after cycling, which can be ascribed to superior ion conductivity of P(AA‐co‐LiAMPS) binder provided by sulfonic anionic group and more integrated electrode structure maintained by noncovalent hydrogen bond.

Two aforementioned *D*_Li_^+^ obtained by CV and GITT are apparent diffusion coefficient of lithium ion, which may not be enough to reflect the actual lithium‐ion diffusion kinetics. Therefore, in order to explore the lithium‐ion diffusion kinetics more accurately, diffusion coefficient of lithium ion was calculated according to the slope of low‐frequency straight line in EIS curves based on the following equation.^[^ ^27^ ^]^

(4)

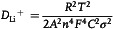

(5) $$Z=R_{s}+\;R_{\text{ct}}+\;\sigma \omega^{-1/2}$$

Generally speaking, *R* represents the gas constant (J (mol•K)^−1^); *T* refers to the thermodynamic temperature (K) during the EIS test; *A* corresponds to the actual area (cm^2^) of the electrode participating in the electrochemical reaction; *n* represents the number of electron‐transfer during lithiation and delithiation process; *F* is the Faraday constant (C mol^−1^); *C* refers to the concentration of Li^+^ in the electrolyte (mol cm^−3^); and *σ* represents the slope of straight line at low frequency region. As shown in Figure 5j–l, *D*_Li_^+^ was calculated according to the slope of low‐frequency straight line. Before cycling, *Z*–*ω* ^−1/2^ curves of SiO_*x*_@P(AA‐co‐LiAMPS) anode exhibits a higher slope than that of SiO_*x*_@PAA anode, which reveals that SiO_*x*_@P(AA‐co‐LiAMPS) anode possesses a lower *D*_Li_^+^ of 4.99 × 10^−17^ cm^2^ s^−1^ than that of SiO_*x*_@PAA anode (14.48×10^−17^ cm^2^ s^−1^) due to inadequate infiltration of electrolyte to the electrode (Table S4, Supporting Information). After 10 cycles, SiO_*x*_@P(AA‐co‐LiAMPS) and SiO_*x*_@PAA anodes exhibit much higher *D*_Li_^+^ which are nearly five orders of magnitude higher than that before cycling, indicating full infiltration of electrolyte to SiO_*x*_ electrodes. At the same time, SiO_*x*_@P(AA‐co‐LiAMPS) anode delivers a *D*_Li_^+^ nearly two times higher than that of SiO_*x*_@PAA anode. As shown in Table S4, Supporting Information, as the cycle continues, SiO_*x*_@P(AA‐co‐LiAMPS) anode exhibits a larger increment of *D*_Li_^+^ than SiO_*x*_@PAA anode, implying more stable SEI and much superior ion conductivity of SiO_*x*_@P(AA‐co‐LiAMPS) anode.

**Figure 6:**
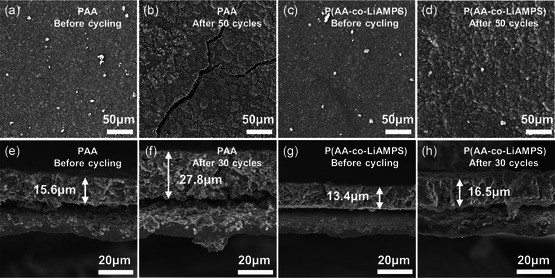
SEM images of a,b) SiO_*x*_@PAA and c,d) SiO_*x*_@P(AA*‐*co‐LiAMPS) anode before cycling and after 50 cycles. Cross‐sectional SEM images of e,f) SiO_*x*_@PAA and g,h) SiO_*x*_@P(AA*‐*co‐LiAMPS) anode before cycling and after 30 cycles.

Scanning electron microscopy (SEM) was conducted to characterize the influence of noncovalent hydrogen bond on the interfacial stability of SiO_*x*_ anode. As illustrated in **Figure** **6**a,c, both pristine SiO_*x*_@PAA anode and SiO_*x*_@P(AA‐co‐LiAMPS) anode exhibit smooth and crack‐free surfaces. Nevertheless, many small cracks obviously appear on the surface of SiO_*x*_@PAA anode only after 5 cycles (Figure S17a, Supporting Information), while SiO_*x*_@P(AA‐co‐LiAMPS) anode still maintains a relatively compact surface (Figure S17b, Supporting Information). After 50 cycles, several deeper and wider cracks can be observed on the surface of SiO_*x*_@PAA anode due to the failure of covalent cross‐linking network between PAA and SiO_*x*_ (Figure 6b), while small cracks existing on SiO_*x*_@P(AA‐co‐LiAMPS) anode before cycling disappear and no new cracks appear (Figure 6d). Pristine SiO_*x*_@PAA anode and SiO_*x*_@P(AA‐co‐LiAMPS) anode exhibit the thicknesses of 15.6 and 13.4 μm before cycling, respectively (Figure 6e,g). After 30 cycles, SiO_*x*_@PAA anode delivers a much thicker cross section of 27.8 μm with an expansion rate of 78.2% (Figure 6f); nevertheless, SiO_*x*_@P(AA‐co‐LiAMPS) anode shows a relatively low thickness of 16.5 μm with a thickness increasing rate of 23.1% (Figure 6h). Such a result demonstrates that higher resilience of P(AA‐co‐LiAMPS) binder has a tremendous effect on avoiding stress concentration, buffering volume variation and maintaining electrode integrity.

**Figure 7:**
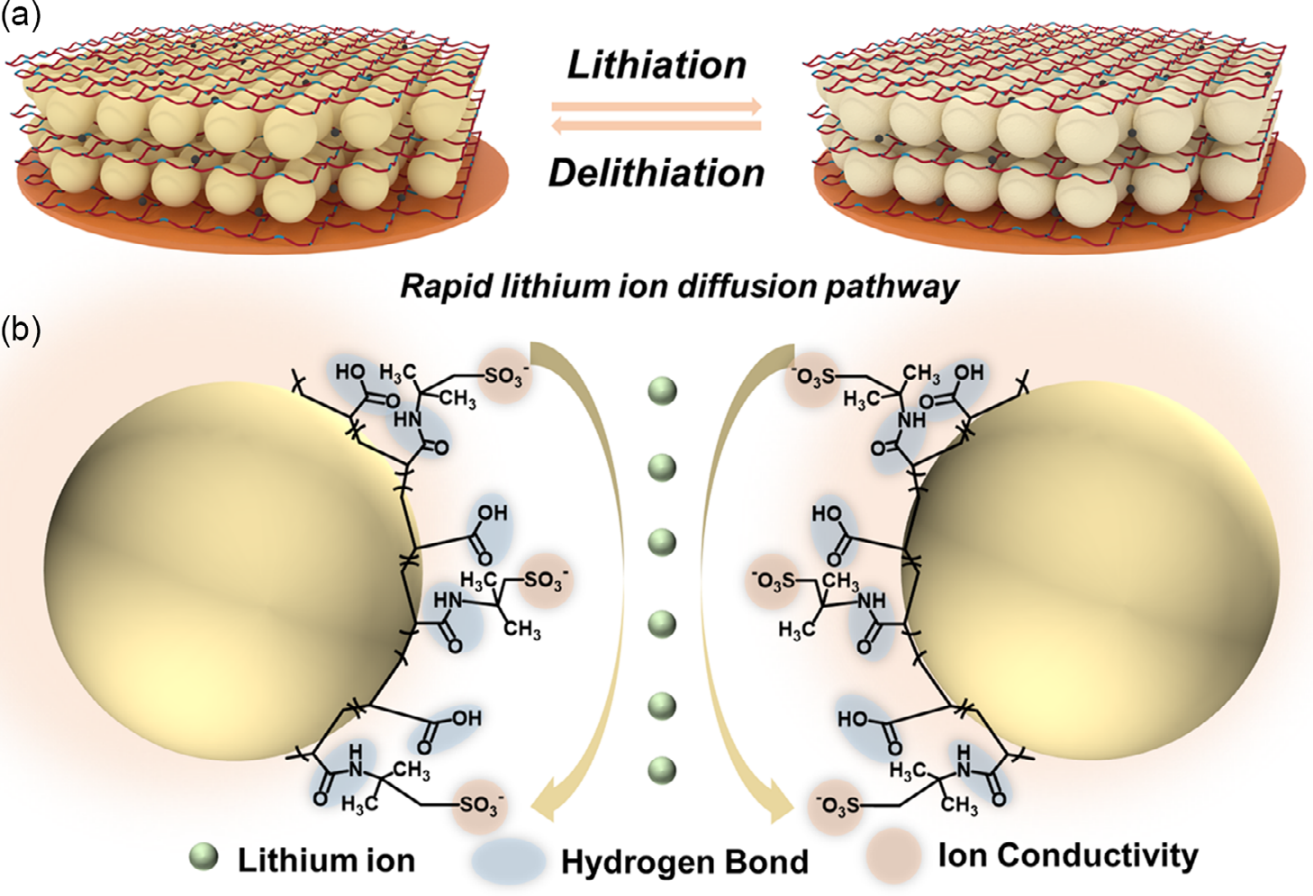
a) Volume expansion and contraction of SiO_*x*_ anode during lithiation and delithiation. b) Rapid lithium‐ion diffusion pathway constructed by ample sulfonic acid anionic groups in P(AA*‐*co‐LiAMPS).

To better understand the improving mechanism of electrochemical performance, a simplified model of lithium‐ion diffusion pathway was established. Considering the micro size of amorphous SiO_*x*_ (Figure S1 and S2a–c, Supporting Information), it is of vital importance to form an effective electrical contact between two SiO_*x*_ microparticles during volume expansion and contraction. High resilience makes the copolymer possible to maintain the structural integrity of SiO_*x*_ anode during volume expansion and contraction, while superior ion conductivity is beneficial to promote uniform Li^+^ transport. As shown in **Figure** **7**a,b, noncovalent hydrogen bond endows P(AA‐co‐LiAMPS) binder with high resilience, effectively dissipating internal stress and avoiding uneven stress concentration, and contributes to accommodate tremendous volume expansion and contraction of SiO_*x*_ anode during lithiation and delithiation. Furthermore, ample sulfonic acid anionic groups construct a rapid lithium‐ion diffusion pathway between SiO_*x*_ microparticles and immensely promote the migration of lithium ion along the polymer chains. Hence, the negatively charged sulfonic acid anionic groups exceedingly decrease the diffusion impedance of Li^+^ through the polymer chains and boost the ion conductivity of P(AA‐co‐LiAMPS) binder. As envisioned, the synergistic effect of high resilience and superior ion conductivity guarantees excellent battery performance.

## 3 Conclusion

To sum up, we proposed a sulfonic acid anionic group‐rich binder with boosted ion conductivity for SiO_*x*_ microparticle anode. High resilience provided by noncovalent hydrogen bond renders effective stress dissipation and avoids uneven stress concentration. Meanwhile, rapid lithium‐ion diffusion pathway constructed by ample sulfonic acid anionic groups accelerates lithium‐ion transportation along the polymer chains and boosts the ion conductivity of binder. Such the ion‐conductive binder with boosted ion conductivity endows SiO_*x*_ microparticle anode with high electrode structural integrity, superior rate performance, and extraordinary cycling stability. Benefiting from such a synergistic effect of constructing lithium‐ion diffusion pathway and alleviating the stress concentration, P(AA‐co‐LiAMPS) binder endows SiO_*x*_ anode with preeminent capacity of 587.8 mAh g^−1^ after 400 cycles at 1C and unprecedented rate performance of 648.6 mAh g^−1^ at 5C. Such a synergistic design strategy provides P(AA‐co‐LiAMPS) binder with a refulgent prospect for high energy density silicon‐based anodes.

## 4 Experimental Section

4.1

4.1.1

##### Synthesis of P(AA‐co‐LiAMPS) Polymer

P(AA‐co‐LiAMPS) was prepared by free radical polymerization of AA (99%, Macklin) and LiAMPS. First, in order to neutralize the sulfonic acid group of AMPS, anhydrous lithium hydroxide (LiOH, 99.9%, Macklin) was added into 2‐acrylamido‐2‐methyl‐1‐propanesulfonic acid (AMPS, 98%, Macklin) solution at the mole ratio of 1:1. Second, AA was dissolved in the prepared LiAMPS solution at the molar ratio of 3:1 (1:1 for P11, 2:1 for P21, and 4:1 for P41). Finally, 0.05 wt% APS (as an initiator, 99.99%, Macklin) was added into the above solution, then the free radical polymerization reaction was conducted at 50 °C for 8 h under the protection of N_2_. The mixture after reaction was followed by freeze drying for 12 h to obtain the P(AA‐co‐LiAMPS) polymer.

Materials characterizations, mechanical characterizations, and electrochemical measurements were performed according to the method reported in our previous work.[^6^, ^12^]

## Conflict of Interest

The authors declare no conflict of interest.

## Supporting information

Supplementary Material

## Data Availability

The data that support the findings of this study are available from the corresponding author upon reasonable request.
