# Supplementary material for: Sulfonic Group Modified Binder Endows Rapid Lithium‐Ion Diffusion for SiO x Microparticle Anode
Source: Small Sci. 2023 Nov 27;4(1):2300133. doi: 10.1002/smsc.202300133 (PMC11935210; doi:10.1002/smsc.202300133)
Supplement: Supplementary file 1 — Supplementary Material [file SMSC-4-2300133-s001.pdf]

Copyright WILEY-VCH Verlag GmbH & Co. KGaA, 69469 Weinheim, Germany, 2021.

Supporting Information

Sulfonic Group Modified Binder Endows Rapid Lithium-Ion Diffusion for SiO_x Microparticle Anode

Zheng Weng, Gang Wu, Jiaqi Li, Ying Zhang, Ruifeng Zhang, Ning Zhang, Xiaohe Liu, Chuankun Jia* and Gen Chen**

Z. Weng, G. Wu, J. Li, Prof. N. Zhang, Prof. G. Chen
School of Materials Science and Engineering, Hunan Provincial Key Laboratory of Electronic Packaging and Advanced Functional Materials, Central South University, Changsha, Hunan 410083, P. R. China
E-mail: ruifeng.zhang@csu.edu.cn (R. Zhang); geenchen@csu.edu.cn (G. Chen)

Prof. C. Jia
Institute of Energy Storage Technology, Changsha University of Science & Technology, Changsha, Hunan 410114, P. R. China
E-mail: jiachuankun@csust.edu.cn (C. Jia)

Dr. Y. Zhang, Prof. X. Liu
Zhongyuan Critical Metals Laboratory and School of Chemical Engineering, Zhengzhou University, Zhengzhou, Henan 450001, P. R. China

Experimental Section

Materials characterizations. The phase structures of SiO_x microparticles and polymer binders were examined by X-ray diffraction (Rigaku MiniFlex 600, Cu Kα radiation, λ=0.154 nm). The morphology information and elementary content of SiO_x microparticles and electrodes were acquired by scanning electron microscope (SEM, MIRA3) with energy-dispersive X-ray spectroscopy (EDX). The surface functional group information of binders and SiO_x microparticles was detected by Fourier transform infrared spectroscopy (FTIR, Nicolet 6700) within 4000 to 400 cm^{-1}. X-ray photoelectron spectroscopy (XPS, Thermo Scientific K-Alpha+) was conducted to investigate the surficial chemistry information of binders. Thermogravimetric analysis (TGA, NETZSCH STA 2500) was carried out to verify the thermostability of the polymer binders under the protection of N_2 from 25 to 500 °C at a heating rate of 10 °C min^{-1}. Differential scanning calorimetry (DSC, NETZSCH DSC 200 F3) was performed to record the glass transition temperature (T_g) of the polymers under the protection of N_2 from -80 to 100 °C at a heating speed of 10 °C min^{-1}. The chemical shifts of ^1H NMR spectra were obtained based on D_2O solvent on a nuclear magnetic resonance (MAS NMR). The molecular weight of polymer was measured by gel permeation chromatograph (GPC, Agilent PL-GPC220).

Mechanical characterizations. Nanoindentation tests (Hysitron TI 950) were performed to examinate the Young's modulus and hardness of polymer binders. The rheological properties of polymer binders were investigated on a rheometer (HAAKE MARS60). The 180° peeling test (stripping rate = 80 mm min^{-1}) was conducted to evaluate the adhesion ability of binders on a universal testing machine (MTS CMT 6103).

Electrochemical measurements. The slurry was prepared by stirring the mixture of SiO_x microparticles (60 wt%, Daejoo Electronic Materials Co., LTD, South Korea), acetylene black (20 wt%, Sinopharm Chemical Reagent Co. Ltd.) and binder (20 wt%) for 8 h. Then the prepared slurry was coating onto a Cu foil following by drying under vacuum for 12 h at 80 °C. Specifically, the mass load of SiO_x was about 1.3 mg cm^{-2}. All electrochemical performances were tested based on 2025 coin-type half cells, which were assembled in an argon-filled glove box (H_2O, O_2 < 0.01 ppm) using 12.5 mm diameter Li foil as the counter electrode, Celgard 2400 film as the separator, commercial 1 M $LiPF_6$ in EC/DEC/EMC (1:1:1, *v*/*v*, DoDoChem) with 10 vol% of FEC (99.9%, DoDoChem) as the electrolyte. All cells were performed charging/discharging test procedure (1C=1300 mAh g^{-1}) on Neware battery test system within a voltage of 0.02-1.5 V after resting for 10 h. Cyclic voltammetry (CV) was tested by electrochemical workstation (Biologic VMP-3 Science Instruments, Claix) within a voltage of 0-2 V (or 0-3 V) at a scanning speed of 0.1 mV s^{-1}.

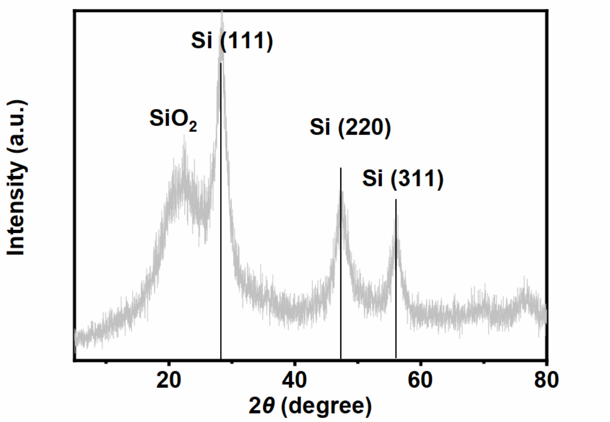

Figure S1. XRD pattern of SiO_x microparticles.

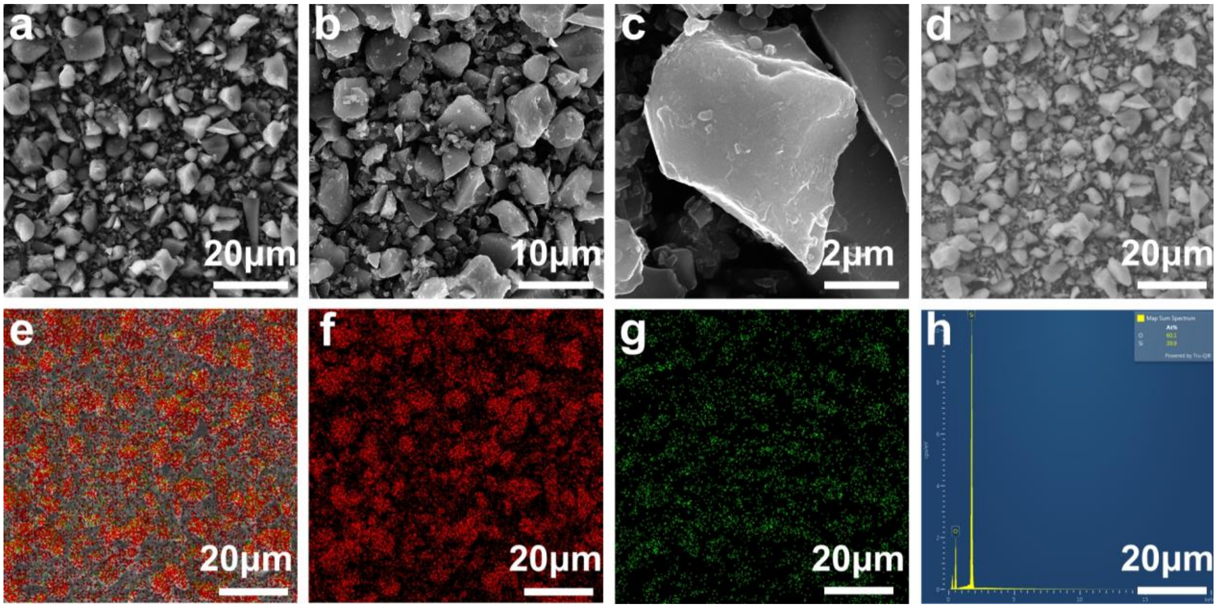

Figure S2. (a-d) SEM image of SiO_x microparticles and (e-g) corresponding elemental mapping. (h) Map Sum Spectrum of SiO_x microparticles.

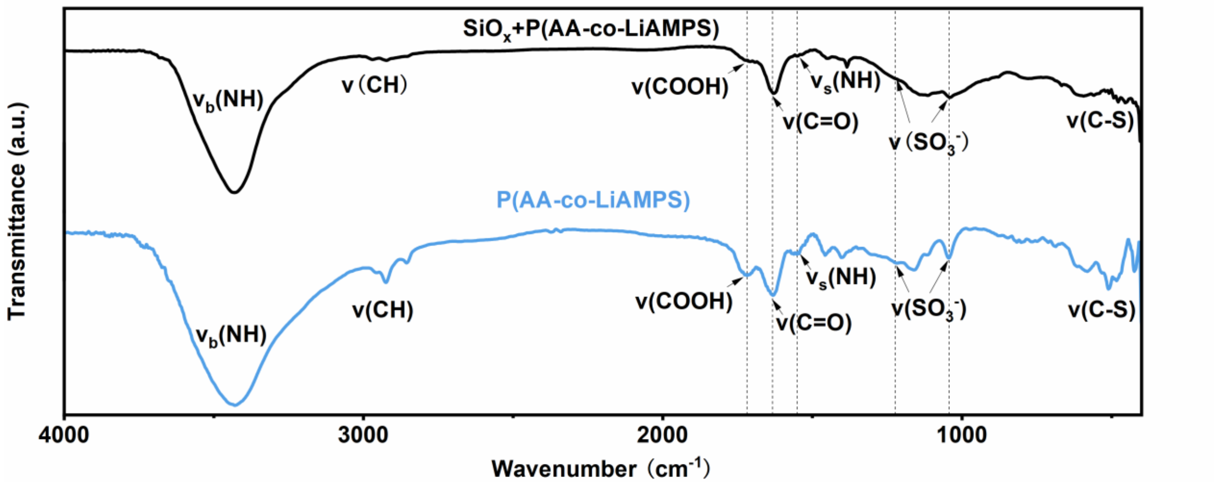

Figure S3. FTIR spectra of SiO_x+P(AA-co-LiAMPS) and P(AA-co-LiAMPS).

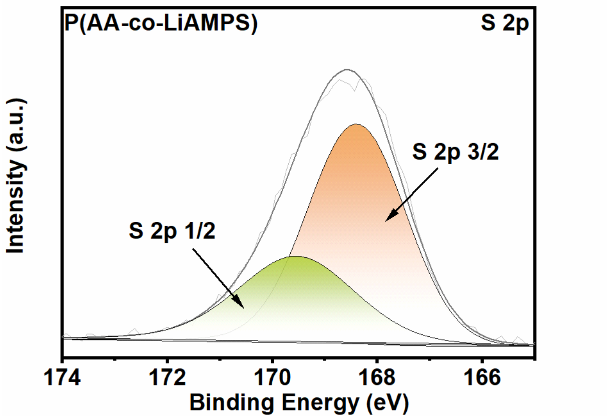

Figure S4. The S 2p high-resolution XPS spectrum of P(AA-co-LiAMPS).

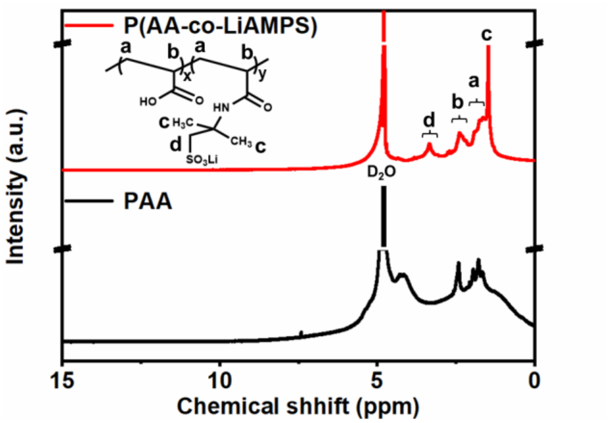

Figure S5. ^{1}H NMR spectra of P(AA-co-LiAMPS) and PAA in D_2O solvent.

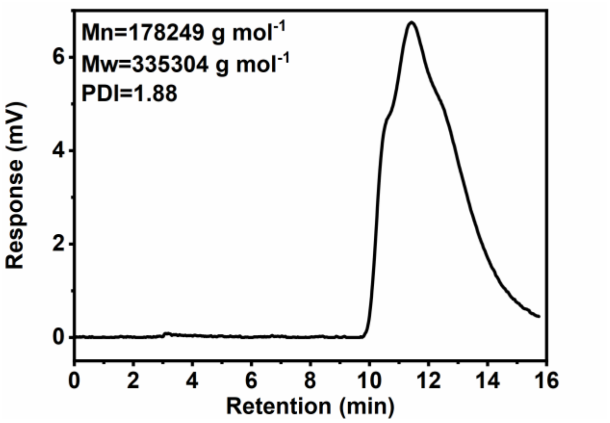

Figure S6. GPC elution trace of P(AA-co-LiAMPS).

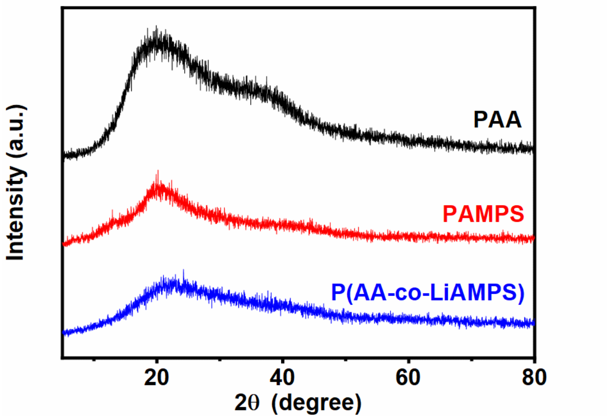

Figure S7. XRD patterns of PAA, PAMPS and P(AA-co-LiAMPS).

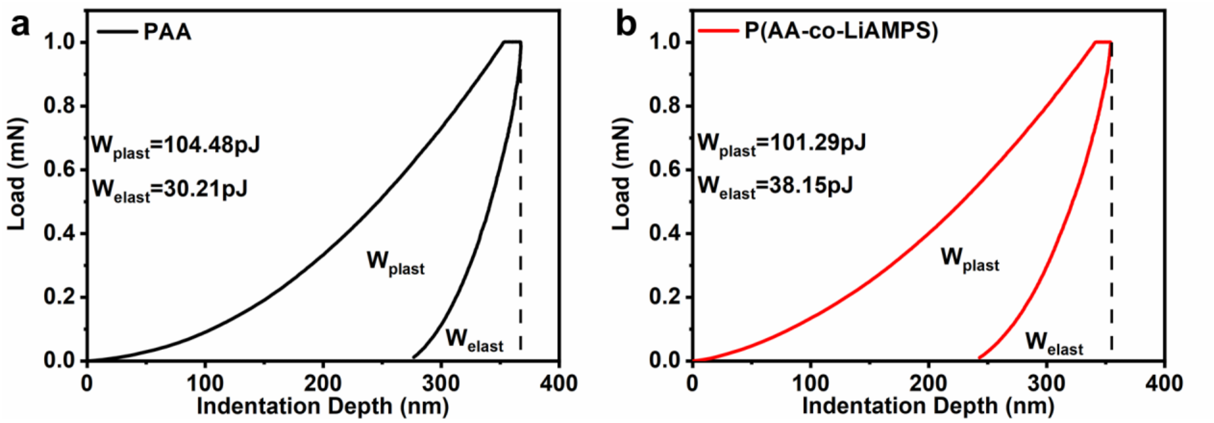

Figure S8. (a) Load-displacement curves of PAA films. (b) Load−displacement curves of P(AA-co-LiAMPS) films.

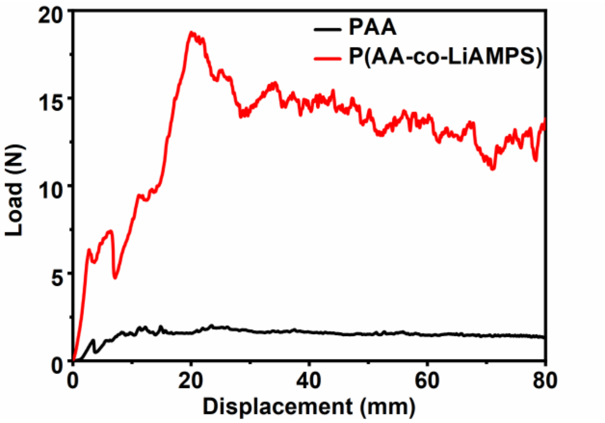

Figure S9. 180° peeling curves of SiO_x electrodes with PAA and P(AA-co-LiAMPS) binders.

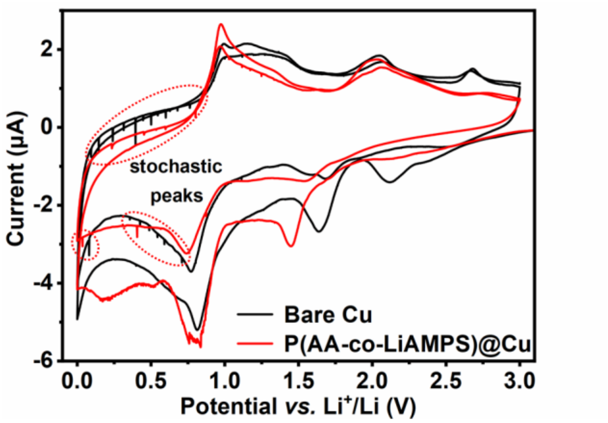

Figure S10. CV profiles of Li||Cu foil half cells with and without polymer binder at the scanning rate of 0.1 mV s^{-1} between 0 and 3 V.

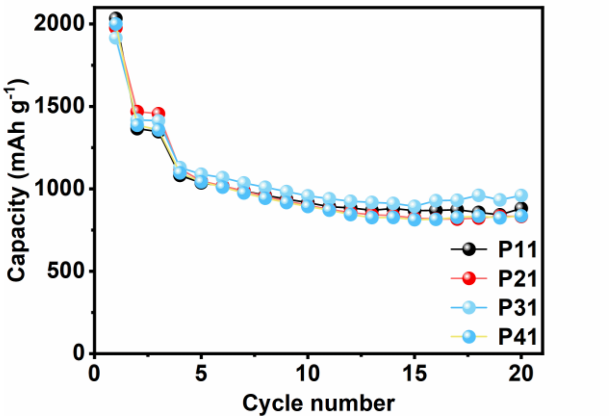

Figure S11. Cycling performances of SiO_x anodes within 20 cycles using P(AA-co-LiAMPS) binders with different mole ratio.

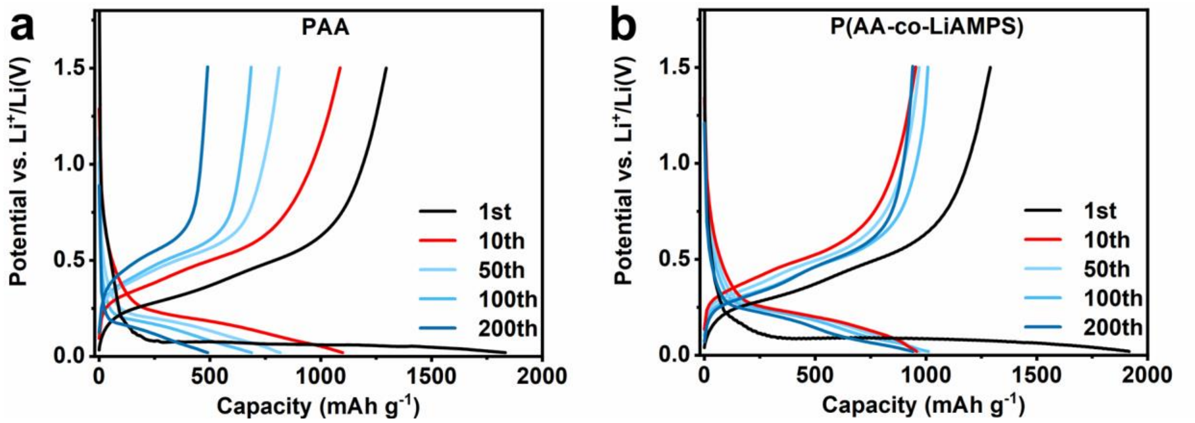

Figure S12. Galvanostatic charge-discharge profiles of (a) SiO_x@PAA anode and (b) SiO_x@P(AA-co-LiAMPS) anode at the 1st, 10th, 50th, 100th and 200th cycle.

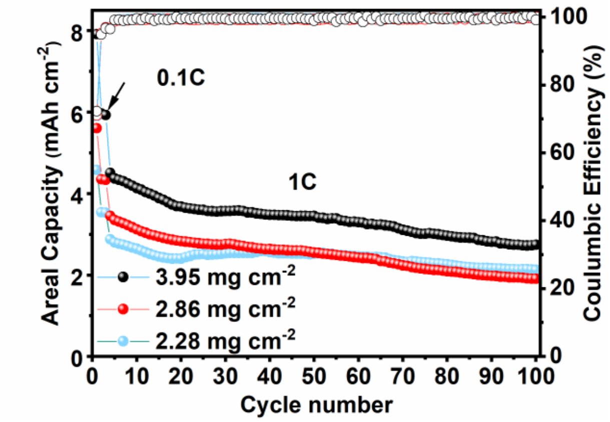

Figure S13. Half-cell cycling performance for SiO_x@P(AA-co-LiAMPS) anodes with different SiO_x loadings at 1 C.

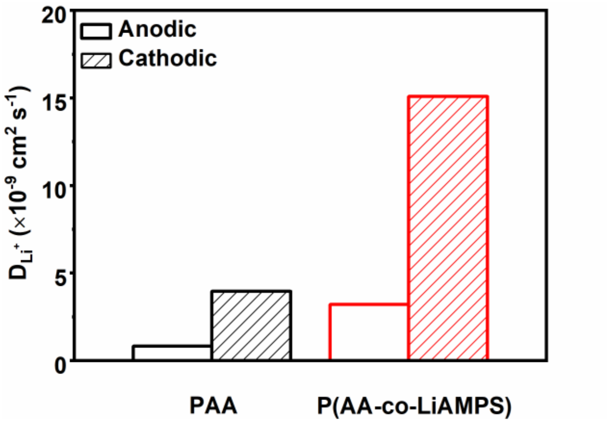

Figure S14. D_{Li}^{+} of SiO_x@PAA and SiO_x@P(AA-co-LiAMPS) anodes calculated from CV curves.

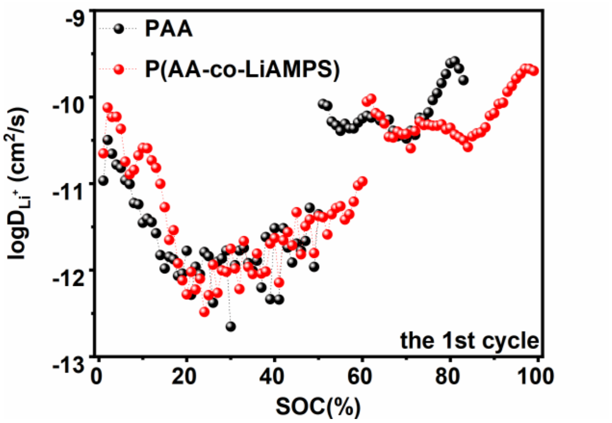

Figure S15. logD_{Li^+}-SOC curves of SiO_x@PAA and SiO_x@P(AA-co-LiAMPS) anodes.

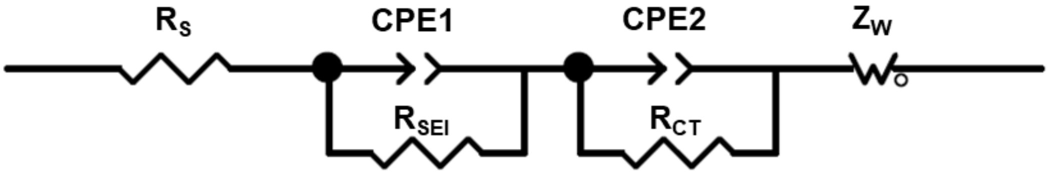

Figure S16. Equivalent circuit model of SiO_x anode.

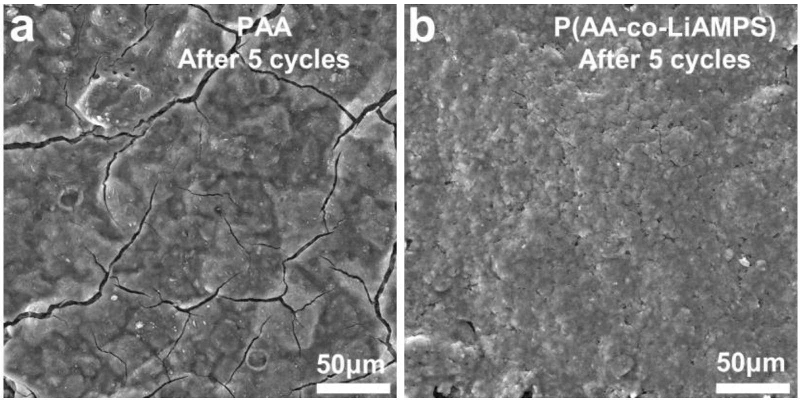

Figure S17. SEM images of a) SiO_x@PAA and b) SiO_x@P(AA-co-LiAMPS) anode after 5 cycles.

Table S1. Calculated content (wt%) and theoretical content (wt%) of Li element in LiAMPS and P(AA-co-LiAMPS).

	Calculated content (wt%)	Theoretical content (wt%)
P(AA-co-LiAMPS)	0.7773	0.8140

Remark: Calculated content (wt%) of Li element was obtained based on the result of Li in P(AA-co-LiAMPS) by ICP. Theoretical content (wt%) of Li element was gained according to the chemical formula of P(AA-co-LiAMPS).

Table S2. Elastic work (W_{elast}), plastic work (W_{plast}) and total work (W_{total}) done by the indenter during this loading process, and corresponding value of nit.

	W_{elast} (pJ)	W_{plast} (pJ)	W_{total} (pJ)	nit (%)
PAA	30.21	104.48	134.69	22.43
P(AA-co-LiAMPS)	38.15	101.29	139.44	27.36

Table S3. Calculated resistances for SiO_x anodes with PAA and P(AA-co-LiAMPS) binders.

	PAA	P(AA-co-LiAMPS)
R_{ct} (Ohm) Before cycling	121.8	78.3
R_{SEI} (Ohm) After 10 cycles	4.7	2.8
R_{ct} (Ohm) After 10 cycles	9.2	5.8
R_{SEI} (Ohm) After 50 cycles	11.7	3.5
R_{ct} (Ohm) After 50 cycles	30.3	13.4

Remark: R_{ct} – electrical transfer resistance of electrode; R_{SEI} − resistance of SEI layer.

Table S4. D_{Li^+} of SiO_x@PAA and SiO_x@P(AA-co-LiAMPS) anodes calculated from EIS curves before cycling and after different cycles.

	Before cycling ($\times10^{-17}$ cm^2 s^{-1})	After 10 cycles ($\times10^{-12}$ cm^2 s^{-1})	After 50 cycles ($\times10^{-12}$ cm^2 s^{-1})
SiO_x@PAA	14.48	3.28	11.67
SiO_x@P(AA-co-LiAMPS)	4.99	7.66	22.24
